# Intimate partner violence and constraints to reproductive autonomy and reproductive health among women seeking abortion services in Bangladesh

**DOI:** 10.1002/ijgo.12070

**Published:** 2016-12-16

**Authors:** Erin Pearson, Kathryn L. Andersen, Kamal Biswas, Rezwana Chowdhury, Susan G. Sherman, Michele R. Decker

**Affiliations:** ^1^ Department of Global Health and PopulationHarvard T.H. Chan School of Public HealthBostonMAUSA; ^2^ Research and Evaluation UnitIpasChapel HillNCUSA; ^3^ Research and Evaluation UnitIpas BangladeshDhakaBangladesh; ^4^ Department of Health, Behavior and SocietyJohns Hopkins Bloomberg School of Public HealthBaltimoreMDUSA; ^5^ Department of Population, Family and Reproductive HealthJohns Hopkins Bloomberg School of Public HealthBaltimoreMDUSA

**Keywords:** Abortion, Bangladesh, Intimate partner violence

## Abstract

**Objective:**

To understand intersections between intimate partner violence (IPV) and other constraints to women's reproductive autonomy, and the influence of IPV on reproductive health.

**Methods:**

A secondary analysis examined cross‐sectional data from a facility‐based sample of women seeking abortion care (for spontaneous or induced abortion) between March 1 and October 31, 2013. Women aged 18–49 years, who received abortion services and selected a short‐acting contraceptive method or no contraception completed an interviewer‐administered survey after treatment. Adjusted prevalence ratios (aPRs) were calculated for associations between IPV experience and potential constraints to reproductive autonomy and health outcomes.

**Results:**

There were 457 participants included in the present analysis and 118 (25.8%) had experienced IPV in the preceding year. IPV was associated with discordance in fertility intentions with husbands/partners and in‐laws, with in‐law opposition to contraception, with perceived religious prohibition of contraception, and with presenting unaccompanied (all *P<*0.05). IPV was also associated with receiving post‐abortion care after an induced abortion compared with accessing legal menstrual regulation, and with the use of medication abortion compared with manual vacuum aspiration (both *P<*0.05).

**Conclusion:**

Intimate partner violence was associated with additional constraints on reproductive autonomy from husbands/partners, in‐laws, and religious communities. Seeking induced abortion unaccompanied and using medication abortion could be strategies to access abortion covertly among women experiencing IPV. Ensuring women's reproductive freedom requires addressing IPV and related constraints.

## Abstract 2

Intimate partner violence was associated with multiple threats to women's reproductive autonomy; the findings identify potential strategies to control fertility in the presence of violence.

## 1 Introduction

Intimate partner violence (IPV) negatively impacts women's health and well‐being and is a major contributor to poor reproductive health.^1^, ^2^ Globally, 30% of ever‐partnered women experience physical or sexual IPV during their lifetimes.^1^ Bangladeshi women are disproportionately affected; an estimated 50%–60% have experienced IPV in their lifetimes, and 30% have experienced such violence in the past year.^3^ IPV is associated with poor reproductive health outcomes in a variety of settings, including Bangladesh,^1^, ^2^, ^4^, ^5^ where IPV is associated with a 50%–60% increase in unwanted pregnancy and a greater than two‐fold increase in the odds of induced abortion,^4^, ^5^ suggesting that women who experience IPV have more limited control over their fertility. Consequently, women seeking induced abortions are a key population for understanding IPV and its influence on women's reproductive health. In Bangladesh, induced abortion is illegal except to save the life of the woman, but menstrual regulation (MR) is a legal uterine evacuation procedure performed using surgical or medication induced‐abortion methods to establish non‐pregnancy up to 10 weeks from an individual's last menstrual period.^6^ Post‐abortion care (PAC) is also widely available in Bangladesh to treat incomplete abortion resulting from induced or spontaneous abortion.^7^

Constraints to women's reproductive autonomy can be considered at multiple levels, including individual perceptions and family factors that affect access to contraception. In the context of IPV, a diminished sense of reproductive agency could prompt women to perceive more limited access to contraception, with IPV victims less able to use contraception effectively owing to refusal by partners and contraceptive sabotage.^8^, ^9^ Discordance in fertility intentions within the family, the most common form being more pro‐natalist preferences on the part of the husband/partner, has been shown to be associated with increased fertility,^10^, ^11^ and could be indicative of pressure from the husband/partner either to continue a pregnancy that a woman wishes to terminate or to terminate a pregnancy that she wishes to continue.^2^, ^12^ Less is known about discordance in fertility intentions of in‐law relatives but recent studies in Côte d'Ivoire have reported high rates of in‐law abuse and reproductive control,^13^ as well as co‐occurrence between IPV and in‐law perpetrated reproductive coercion,^14^ highlighting the influence of in‐laws and suggesting that multiple layers of reproductive control can be present within the family. Beyond discordance in fertility intentions, women can have limited power within the family, including constrained decision‐making authority, mobility, and direct opposition to contraceptive use. A study performed in two rural areas of Bangladesh^15^ demonstrated that women's autonomy was inversely related to IPV experience; women who travelled unaccompanied, who were involved in household decision‐making, and who had control over financial resources were less likely to experience IPV.

The aim of the present study was to examine IPV experiences in the past year and other potential constraints to reproductive autonomy among women utilizing abortion services in Bangladesh, with the goal of identifying the influence of IPV on reproductive health outcomes.

## 2 Materials and Methods

The present study was a cross‐sectional analysis of data collected under a prospective parent study that aimed to understand short‐acting post‐abortion contraceptive use among women using public‐sector abortion services in Bangladesh (unpublished data). The parent study collected data between March 1 and October 31, 2013, using a stratified one‐stage cluster sampling approach. The sampling frame consisted of 47 public‐sector facilities that were participating in an intervention to improve the quality of abortion care; 16 were randomly selected for inclusion using probability proportional to size sampling within facility‐type strata. Women were eligible for inclusion in the parent study if they were presenting for abortion services (MR or PAC), were aged 18–49 years, and if they selected to use pills, injectable contraception, or condoms as post‐abortion contraception, or if they chose no contraception. The parent study was focused on women who chose to use short‐acting contraceptive methods or no contraception; women who selected long‐acting or permanent contraceptive methods were ineligible. The parent study protocol received ethical approval from the Bangladesh Medical Research Council in Dhaka, Bangladesh, and the Allendale Investigational Review Board in the USA; written informed consent was obtained from all participants.

After recovering from abortion procedures, eligible women were introduced to female research assistants who were posted at each facility during all available clinic hours. Research assistants confirmed participants' eligibility and obtained written informed consent before participants completed a 30‐minute interviewer‐administered survey. Consistent with international ethical standards for research into violence against women,^16^ interviewers confirmed participant privacy before proceeding to ask questions regarding experiences of violence; this questionnaire section was skipped if privacy could not be attained. Participants with incomplete IPV data were not included in the present analysis.

The exposure of interest, IPV experience during the preceding year, included experience of either physical or sexual violence that was perpetrated by the woman's husband or sexual partner. Standard questions from the Bangladesh Demographic and Health Survey were used;^17^ these are based on the validated and widely used Conflict Tactics Scales (CTS2):^18^ “In the past year has your husband/partner hit, kicked, slapped or otherwise physically hurt you?” and, “In the past year, has your husband/partner physically forced you to have sexual intercourse with him even when you did not want to?”

The outcomes assessed were individual perceptions about access to contraception, discordance in fertility intentions within the family, family‐level constraints to reproductive autonomy, and measures of women's reproductive health. Perceptions about access to contraception were assessed using three yes or no questions: (1) “Do you think that it is too difficult to obtain family planning methods, or that you would have to travel too far to obtain a method?”; (2) “Do you think that it is too expensive to obtain family planning methods?”; and (3) “Do you think that family planning methods are inconvenient to use?” Discordance in fertility intentions was assessed both for intentions regarding the index pregnancy (ending in abortion) and future pregnancies. Intentions regarding the index pregnancy were assessed by asking, “Right before you became pregnant, did you want to become pregnant then, did you want to wait until later, did you not want to have any (more) children, or did you not think about it?”.^19^ Women were asked the same question about their husbands'/partners' and in‐law relatives' intentions. Future fertility intentions were assessed by asking women whether they wanted a/another child in the future, and they were asked the same question about their husbands'/partners' preferences. Discordance was constructed by ordering family member intentions from the highest to lowest desire for fertility, and creating three categories relative to a participant's intentions:^20^ concordant, discordant‐higher, and discordant‐lower. Opposition to contraceptive use among family members, being accompanied to healthcare facilities for abortion, and household decision‐making were also assessed. The reproductive health outcomes examined were history of MR, type of treatment received (MR or PAC for induced abortion, or PAC for spontaneous abortion), and procedure type (manual vacuum aspiration, medication abortion, or dilation and curettage).

Socio‐demographic characteristics were presented for the full study population and by IPV experience; F‐tests from simple logistic regression models were used to test bivariate associations. Each potential constraint to reproductive autonomy and reproductive health outcome was presented across the full sample and by IPV experience. Adjusted prevalence ratios (aPRs) were calculated using multinomial logistic regression models for categorical outcomes and generalized linear models using log‐binomial maximum likelihood estimators for dichotomous outcomes. The Poisson distribution was specified if the model failed to converge using the binomial distribution, a conservative approach that was expected to result in valid point estimates with confidence intervals wider than those from log‐binomial estimates.^21^ All multivariable models were adjusted for age, education, and rural‐to‐urban migrant status. Univariate imputation was used to generate ten imputations for each outcome measure with missing data, up to a maximum of 8% missing. Stata/SE version 14.0 (StataCorp, College Station, TX, USA) was used to analyze the imputed dataset, accounting for the complex survey design, and *P<*0.05 was considered statistically significant.

## 3 Results

A total of 555 eligible women were approached for participation in the parent study and 498 were enrolled. There were 41 women (8%) excluded from the present analysis owing to missing IPV‐experience data. Residence location was the only variable that was associated with missing IPV data owing to clustering in several facilities where privacy was difficult to obtain. This resulted in a sample size of 457 women with complete IPV data.

The mean age of participants was 27 years (range 18–45), 374 (81.8%) had at least one child, 251 (54.9%) had secondary or higher education, 406 (88.9%) were Muslim, and almost all were married at the time of the study (Table 1). Of the participants, 269 (58.9%) resided in urban areas and 116 (25.4%) were rural‐to‐urban migrants. The mean age of participants' husbands was 35 years (range 20–60), and 248 (54.3%) had secondary or higher education. In total, 118 (25.8%) participants had experienced IPV during the preceding year; the incidence of IPV differed across rural‐to‐urban migrant status and division of residence.

**Table 1:** Baseline characteristics.^a^

Variable	All study participants (n=457)	No experience of IPV in the preceding 12 mo (n=339)	Experienced IPV in the preceding 12 mo (n=118)	*P* value
Age, y	27.3±0.46	27.1±0.57	27.8±0.67	0.434
Husband/partner's age, y	34.8±0.70	34.8±0.90	35.1±0.78	0.784
Education completed				0.357
None	66 (14.4)	47 (71.2)	19 (28.8)	
Primary	140 (30.6)	96 (68.6)	44 (31.4)	
Secondary or higher	251 (54.9)	196 (78.1)	55 (21.9)	
Husband/partner's education				0.416
None	78 (17.0)	54 (69.2)	24 (30.8)	
Primary	131 (28.7)	95 (72.5)	36 (27.5)	
Secondary or higher	248 (54.3)	190 (76.6)	58 (23.4)	
Religion				0.983
Islam	406 (88.9)	301 (74.1)	105 (25.9)	
Hinduism	50 (10.9)	37 (74.0)	13 (26.0)	
Buddhism	1 (0.2)	1 (100)	0	
Marital status				NA
Married	456 (99.8)	339 (74.3)	117 (25.7)	
Formerly married	1 (0.2)	0	1 (100)	
No. of children				0.941
0	83 (18.2)	63 (75.9)	20 (24.1)	
1–2	257 (56.2)	191 (74.3)	66 (25.7)	
≥3	117 (25.6)	85 (72.6)	32 (27.4)	
Household structure				0.739
Nuclear	253 (55.4)	185 (73.1)	68 (26.9)	
Extended	204 (44.6)	154 (75.5)	50 (24.5)	
Husband/partner's residence				0.115
Lives with husband/partner	420 (91.9)	318 (75.7)	102 (24.3)	
Does not live with husband/partner	37 (8.1)	21 (56.8)	16 (43.2)	
Residence				0.102
Urban	269 (58.9)	187 (69.5)	82 (30.5)	
Rural	188 (41.1)	152 (80.9)	36 (19.1)	
Rural‐to‐urban migrant				0.007
Yes	116 (25.4)	75 (64.7)	41 (35.3)	
No	341 (74.6)	264 (77.4)	77 (22.6)	
Division of residence				0.041
Dhaka	233 (51.0)	157 (67.4)	76 (32.6)	
Sylhet	117 (25.6)	103 (88.0)	14 (12.0)	
Chittagong	61 (13.3)	47 (77.0)	14 (23.0)	
Rajshahi	46 (10.1)	32 (69.6)	14 (30.4)	

Abbreviations: IPV, intimate partner violence; NA, not applicable.

a Values are given as mean±standard error or number (percentage), unless indicated otherwise.

Experiencing IPV in the preceding year was associated with multiple other constraints to reproductive autonomy (Table 2). Women who had experienced IPV in the past year had a higher prevalence of reporting that contraception was too difficult to obtain compared with not too difficult (aPR 1.81, 95% confidence interval [CI] 1.05–3.09), and of reporting that contraception was inconvenient to use compared with not inconvenient (aPR 1.73, 95% CI 1.01–2.95). Women who had experienced IPV had a higher prevalence of discordance in fertility intentions with their husbands/partners (aPR 2.41, 95% CI 1.46–3.98) and with in‐law relatives (aPR 1.98, 95% CI 1.44–2.74) regarding the index pregnancy, compared with women reporting concordant intentions. The discordant‐lower category of fertility intentions was excluded from the analysis owing to the small number of responses in this category. In addition, women who had experienced IPV had a higher prevalence of reporting discordance in future pregnancy intentions with their partners (aPR 2.92, 95% CI 1.62–5.26), indicating that they were more likely to perceive that their husbands/partners wanted more children when they did not. A higher prevalence of opposition to contraceptive use by in‐law relatives (aPR 3.21, 95% CI 1.50–6.87) and of reporting religious prohibitions to contraceptive use (aPR 1.63, 95% CI 1.09–2.44) were demonstrated among participants who had experienced IPV in the previous year. Only 44 (9.6%) participants attended healthcare facilities alone, but a higher prevalence of presenting unaccompanied compared with being accompanied by their husbands/partners was recorded among women who had experienced IPV in the preceding year (aPR 2.25, 95% CI 1.05–4.85).

**Table 2:** Multinomial logistic regression models of IPV during the preceding year and potential constraints to reproductive autonomy (n=457).^a^

Outcome	All study participants (n=457)	No experience of IPV in the preceding 12 mo (n=339)	Experienced IPV in the preceding 12 mo (n=118)	Adjusted prevalence ratio (95% confidence interval)
Perceived access to contraception				
Obtaining contraception^b^				
Not too difficult	401 (90.0)	302 (91.7)	99 (85.1)	1.00
Too difficult	44 (10.0)	27 (8.3)	17 (14.9)	1.81 (1.05–3.09)
Contraceptive expense^b^				
Not too expensive	384 (88.1)	291 (90.9)	93 (80.1)	1.00
Too expensive	52 (11.9)	29 (9.1)	23 (19.9)	2.04 (0.74–5.61)
Convenience^b^				
Not inconvenient	333 (78.6)	257 (82.1)	76 (68.6)	1.00
Inconvenient	89 (21.4)	54 (17.9)	35 (31.4)	1.73 (1.01–2.95)
Discordance in fertility intentions				
Husband/partner's relative intentions regarding index pregnancy^c^				
Concordant	402 (90.3)	309 (93.1)	93 (82.3)	1.00
Discordant‐higher	43 (9.7)	23 (6.9)	20 (17.7)	2.41 (1.46–3.98)
In‐laws' relative intentions regarding index pregnancy^d^				
Concordant	200 (71.9)	158 (77.5)	42 (56.8)	1.00
Discordant‐higher	78 (28.1)	46 (22.5)	32 (43.2)	1.98 (1.44–2.74)
Husband/partner's relative intentions regarding future pregnancies^e^				
Concordant	428 (94.3)	324 (96.1)	104 (88.9)	1.00
Discordant‐higher	26 (5.7)	13 (3.9)	13 (11.1)	2.92 (1.62–5.26)
Household decision making				
Contraceptive‐use decision making				
Not involved	33 (7.2)	22 (6.5)	11 (9.3)	1.00
Involved	424 (92.8)	317 (93.5)	107 (90.7)	0.97 (0.90–1.05)
Participant healthcare decision making				
Not involved	76 (16.6)	51 (15.0)	25 (21.2)	1.00
Involved	381 (83.4)	288 (85.0)	93 (78.8)	0.92 (0.82–1.04)
Opposition to contraceptive use				
Husband/partner opposition^b^				
Not opposed	422 (95.7)	319 (96.6)	103 (93.0)	1.00
Opposed	18 (4.3)	11 (3.4)	7 (7.0)	2.09 (0.58–7.40)
In‐laws' opposition^f^				
Not opposed	293 (92.1)	228 (95.0)	65 (83.3)	1.00
Opposed	25 (7.9)	12 (5.0)	13 (16.7)	3.21 (1.50–6.87)
Religious prohibition^b^				
Does not prohibit	319 (76.1)	245 (79.1)	74 (67.4)	1.00
Prohibits contraceptive use	100 (23.9)	63 (20.9)	37 (32.6)	1.63 (1.09–2.44)
Accompaniment to the healthcare facility				
Accompaniment for abortion procedure				
Husband	246 (53.8)	193 (56.9)	53 (44.9)	1.00
None/alone	44 (9.6)	26 (7.7)	18 (15.3)	2.25 (1.05–4.85)
Someone else	167 (36.6)	120 (35.4)	47 (39.8)	1.42 (0.74–2.75)

Abbreviation: IPV, intimate partner violence.

a Values are given as number (percentage) unless indicated otherwise.

b Multiple imputation variable. Original number (i.e. n≠457) and imputed percent presented.

c Discordant‐lower was excluded from the analysis due to the small sample size (n=12).

d Discordant‐lower was excluded from the analysis due to the small sample size (n=8). In addition, women who did not know their in‐laws' intentions regarding the index pregnancy were excluded.

e Discordant‐lower was excluded from the analysis due to the small sample size (n=3).

f Women who did not know whether their in‐laws opposed contraceptive use were excluded.

Associations were also observed between having experienced IPV in the previous year and reproductive health outcomes (Table 3). A higher prevalence of having a history of MR (aPR 1.49, 95% CI 1.08–2.07) compared with not was recorded among women who had experienced IPV. A higher prevalence of reporting having received PAC for an induced abortion compared with having undergone MR (aPR 2.39, 95% CI: 1.01–5.70), and of undergoing medication abortion compared with manual vacuum aspiration (aPR 2.38, 95% CI 1.57–3.62), was demonstrated among women who had experienced IPV.

**Table 3:** Multinomial logistic regression models of IPV during the preceding year and reproductive health outcomes (n=457).^a^

Outcome	All study participants (n=457)	No experience of IPV in the preceding 12 mo (n=339)	Experienced IPV in the preceding 12 mo (n=118)	Adjusted prevalence ratio (95% confidence interval)
History of MR				
No	332 (72.6)	258 (76.1)	74 (62.7)	1.00
Yes	125 (27.4)	81 (23.9)	44 (37.3)	1.49 (1.08–2.07)
Type of abortion treatment received				
MR	270 (59.1)	209 (61.6)	61 (51.7)	1.00
PAC for induced abortion	74 (16.2)	87 (25.7)	31 (26.3)	2.39 (1.01–5.70)
PAC for spontaneous abortion	113 (24.7)	43 (12.7)	26 (22.0)	0.93 (0.38–2.28)
Procedure type				
MVA	340 (74.4)	258 (76.1)	82 (69.5)	1.00
MA	35 (7.7)	20 (5.9)	15 (12.7)	2.38 (1.57–3.62)
D&C	82 (17.9)	61 (18.0)	21 (17.8)	0.99 (0.39–2.52)

Abbreviations: IPV, intimate partner violence; MR, menstrual regulation; PAC, post‐abortion care; MVA, manual vacuum aspiration; MA, medication abortion; D&C, dilation and curettage.

a Values are given as number (percentage) unless indicated otherwise.

## 4 Discussion

The present study found that over 25% of women seeking abortion care reported experiencing IPV in the preceding year; in turn, this was associated with other potential constraints to reproductive autonomy and reproductive health outcomes. The findings suggest that husbands/partners, in‐laws, and religious communities play a role in women's reproductive lives, with women who experienced IPV reporting discordance in fertility intentions within the family and opposition to contraceptive use. Associations between IPV with PAC services for induced abortion rather than MR suggests that women who experienced IPV could be more likely to attempt induced abortion illegally outside the healthcare system. The findings suggest that women experiencing IPV could be taking active steps to control fertility when faced with IPV, including seeking abortion services unaccompanied and selecting medication abortion as an induced abortion procedure, which can be used to simulate spontaneous abortion and terminate pregnancy covertly.^2^

Women who experienced IPV perceived contraception to be difficult to access. Despite widespread availability of contraceptive methods such as oral contraceptive pills having been reported at the community level,^22^ IPV was associated with contraception being reported as being too difficult to obtain and inconvenient to use. These findings could indicate that women who experience violence lack reproductive agency, making contraceptive use and access difficult. Alternatively, IPV could impact access to contraception more directly if women experience restricted mobility or are subject to reproductive coercion. Women experiencing IPV were also more likely to have a history of MR, suggesting barriers to contraceptive use over time. More research is needed to understand the mechanisms through which women experiencing violence perceive impeded access to contraception.

Intimate partner violence was also associated with discordance in fertility intentions within the family and opposition to contraceptive use. Women who experience IPV could also experience fertility pressure or pregnancy coercion from husbands/partners and in‐laws. Ethnographic work in Bangladesh has demonstrated the interplay between fulfilling in‐laws' role expectations and IPV;^23^ expectations regarding childbearing could contribute to abuse experiences. The present findings suggested that women who experienced IPV faced pressure for childbearing that could extend to experiences of abuse or reproductive coercion from their in‐laws in addition to their husbands/partners, as has been demonstrated in Côte d'Ivoire.^14^ IPV was also associated with a perceived religious prohibition on contraception. Unlike prior studies in Bangladesh,^5^, ^15^ the present study recorded equivalent rates of IPV in the preceding year among Muslim and Hindu groups (25.9% and 26.0%, respectively). However, the findings suggested higher rates of violence among those who perceive religious prohibitions to contraceptive use, which could indicate higher rates of violence among those who were more religiously conservative or who lived in more religiously conservative areas.

The present study also provided potential insights into strategies used by women experiencing IPV to control their fertility. Women who reported IPV were more likely to attend unaccompanied, to seek care for PAC for an induced abortion, and to select medication abortion as an induced abortion procedure; all of which could indicate a propensity for accessing induced‐abortion services covertly, without the knowledge of their families. The higher prevalence of accessing PAC for induced abortions, compared with attending for care for legal MR could indicate that women experiencing IPV were more likely to access induced‐abortion services outside the healthcare system; however, the present study was not able to ascertain the safety or quality of care, which could range from induction using instruments such as sticks or roots to accessing medication abortion drugs through a pharmacy. The finding that women who reported IPV were more likely to select an induced medication abortion compared with manual vacuum aspiration was in line with reported use of induced medication abortion to simulate spontaneous abortion without the knowledge of abusive spouses.^2^

Overall, the present findings suggested that IPV intersects with other constraints to reproductive autonomy imposed by a husband/partner, in‐laws, and religious communities. The results suggested that women could respond to these threats by seeking multiple MR procedures over time and accessing induced abortion services covertly. The findings should be viewed in the context of macro‐level indicators such as the gender inequality index. In 2013, Bangladesh ranked 115 out of 151 countries, with a gender inequality index value of 0.529,^24^ suggesting a broader context of gender‐based inequality that could interact synergistically with the constraints observed herein. The present study sheds light on the multiple threats women face; this suggests that interventions are needed at the household and community levels to improve women's reproductive autonomy, and ultimately gender equality in Bangladesh and other settings where constraints on freedom compromise reproductive health.

The results of the present study should be viewed in light of the limitations. The study focused on an abortion care‐seeking population, and generalizing to the broader population of women experiencing IPV in Bangladesh could be inappropriate. The sample was limited to facilities participating in an intervention to improve the quality of abortion care in more urban areas of Bangladesh, and women younger than 18 years and those selecting long‐acting and permanent contraceptive methods were excluded. Consequently, the findings might not be generalizable to all patients seeking abortion services in Bangladesh. The findings were also limited by the available IPV data because information on the frequency and severity of violence and reproductive coercion was lacking. Finally, family‐member fertility intentions were based on the respondent's report, and could reflect family discord rather than true discordance in fertility preferences.

Women experiencing IPV face additional constraints to reproductive autonomy, and IPV was associated with reproductive health outcomes, including accessing PAC for induced abortions compared with legal MR services. Further research is needed to improve the understanding of women's perspectives on access to contraception and potential strategies to control fertility in the context of IPV. Preliminary findings from the present study suggest that interventions are needed at multiple levels to mitigate the impact of violence and lack of reproductive autonomy on women's reproductive health.^2^ Seeking abortion care unaccompanied and accessing induced medication abortions could be strategies used to control fertility covertly in the context of violence, and facilities should ensure that the full range of procedures, including medication abortion, are available to women. At the community level, husbands/partners, in‐laws, and religious communities should be engaged to improve access to reproductive health services, including safe, legal MR care.

## Author Contributions

EP, KLA, KB, and RC designed the study with input from MRD. KB and RC provided input on the study tools and managed data collection. EP led the analysis and writing with input from all authors. All authors reviewed and approved the final version of the manuscript.

## Conflict of Interest

The authors have no conflicts of interest.
